# Estimation of renal function by CKD-EPI versus MDRD in a cohort of HIV-infected patients: a cross-sectional analysis

**DOI:** 10.1186/s12882-017-0470-4

**Published:** 2017-02-10

**Authors:** M. P. Cristelli, F. Cofán, N. Rico, J. C. Trullàs, C. Manzardo, F. Agüero, J. L. Bedini, A. Moreno, F. Oppenheimer, J. M. Miro, the CKD-H. Clinic Investigators, Fritz Dieckman, Aleix Cases, Esteban Poch, Esteban Martinez, José Luís Blanco, Felipe García, Josep Mallolas, Josep María Gatell

**Affiliations:** 1 Kidney Transplant Division, Hospital do Rim (São Paulo, Brazil), Universidade Federal de São Paulo, São Paulo, Brazil; 2 Nephrology and Renal Transplantation Service, Hospital Clinic – IDIBAPS, University of Barcelona, Barcelona, Spain; 3 Core Laboratory, Biomedical Diagnosis Centre, Hospital Clínic, Barcelona, Spain; 4 Internal Medicine Service, Hospital de Olot, Girona, Spain; 5 Medical Sciences Department, University of Girona, Girona, Spain; 6 Infectious Diseases Service, Hospital Clinic – IDIBAPS, University of Barcelona, Barcelona, Spain

**Keywords:** HIV-infection, Chronic kidney disease, Glomerular filtration rate estimates

## Abstract

**Background:**

Accurately determining renal function is essential for clinical management of HIV patients. Classically, it has been evaluated by estimating glomerular filtration rate (eGFR) with the MDRD-equation, but today there is evidence that the new Chronic Kidney Disease Epidemiology Collaboration (CKD-EPI) equation has greater diagnostic accuracy. To date, however, little information exists on patients with HIV-infection. This study aimed to evaluate eGFR by CKD-EPI vs. MDRD equations and to stratify renal function according to KDIGO guidelines.

**Methods:**

Cross-sectional, single center study including adult patients with HIV-infection.

**Results:**

Four thousand five hundred three patients with HIV-infection (864 women; 19%) were examined. Median age was 45 years (IQR 37–52), and median baseline creatinine was 0.93 mg/dL (IQR 0.82–1.05). A similar distribution of absolute measures of eGFR was found using both formulas (*p* = 0.548). Baseline median eGFR was 95.2 and 90.4 mL/min/1.73 m^2^ for CKD-EPI and MDRD equations (*p* < 0.001), respectively. Of the 4503 measurements, 4109 (91.2%) agreed, with a kappa index of 0.803. MDRD classified 7.3% of patients as “mild reduced GFR” who were classified as “normal function” with CKD-EPI. Using CKD-EPI, it was possible to identify “normal function” (>90 mL/min/1.73 m^2^) in 73% patients and “mild reduced GFR” (60–89 mL/min/1.73 m^2^) in 24.3% of the patients, formerly classified as >60 mL/min/1.73 m^2^ with MDRD.

**Conclusions:**

There was good correlation between CKD-EPI and MDRD. Estimating renal function using CKD-EPI equation allowed better staging of renal function and should be considered the method of choice. CKD-EPI identified a significant proportion of patients (24%) with mild reduced GFR (60–89 mL/min/1.73 m^2^).

## Background

Chronic kidney diseases (CKD) have become one of the leading causes of mortality among patients with HIV infection, and are often associated with comorbidities that affect kidney function, such as diabetes mellitus and hypertension [1–4]. The reported prevalence of renal diseases ranges from 3 to 24% [4–8], depending on the definition of renal disease, study population, and access to health services. Furthermore, although the widespread use of antiretroviral therapy (ARV) has decreased the incidence of HIV-associated nephropathy, the overall prevalence of renal disease continues to increase among people living with HIV [9].

An accurate assessment of renal function in the HIV-infected population is essential, because HIV treatment with ARV involves complex drug regimens often accompanied by significant side effects and drug interactions. A significant number of these drugs undergo renal elimination and demand dose-adjustments according to kidney function [10].

Methods based on the clearance of exogenous markers, like inulin, ^51^Cr-EDTA and iohexol, are the gold standard for glomerular filtration rate (GFR) measurement, but are cumbersome and infrequently used in clinical settings [11]. Numerous equations used to estimate creatinine clearance or GFR have been used over the past few decades. Two of the most commonly used equations (Cockroft-Gault [CG] and Modification of Diet in Renal Disease [MDRD]) are derived from persons with GFR ≤90 mL/min/1.73 m^2^, have not been validated among persons with normal kidney function, and tend to underestimate higher GFRs [12]. Meanwhile, the CKD-EPI (CKD Epidemiology Collaboration) equation has been evaluated in several populations and become the most accurate GFR estimating equation [13–15]. Recently, the CKD-EPI equation has been recommended as the first-choice method to assess renal function by the 2016 European AIDS Clinical Society (EACS) Guidelines [16].

This study aimed to analyze eGFR in a large cohort of patients with HIV infection followed-up in a single center and to evaluate the renal function estimation obtained by the CKD-EPI versus the MDRD equation, also stratifying patients according to different degrees of chronic kidney disease.

## Methods

This was an observational, cross-sectional, retrospective, single center study. The study project was reviewed and approved by the Institutional Review Board (CEIC Hospital Clinic i Provincial, Barcelona, Spain, IRB# 2014/1080). Eligible patients were all adult HIV-1-infected patients (>18 years old) with at least one routine visit during the calendar year of 2014. Patients were excluded if they had not had at least two measurements of serum creatinine during the year 2014 and/or if they had had a diagnosis of end stage renal disease, dialysis and/or transplantation before the diagnosis of HIV infection. Patients were considered as having hypertension, diabetes and/or dyslipidemia if they were receiving specific treatment for these conditions.

All available serum creatinine values were obtained from the hospital electronic patient record system and were converted into eGFR, using age, gender and the CKD-EPI and MDRD equations. We did not consider the African-American coefficient factor as applicable to black patients from Africa, Europe and the Antilles [17], because this information is not available in the electronic medical records, although the prevalence of black patients in our institution, regardless their HIV-status, is very low (<3%).

Impaired renal function (eGFR <90 mL/min/1.73 m^2^) was confirmed with a second measurement at least 3 months apart from the first “baseline” creatinine. We used clinical guidelines from the US National Kidney Foundation’s Kidney Disease Outcome Quality Initiative (K/DOQI) to grade renal impairment [18]. In this study, eGFR below 60 mL/min/1.73 m^2^ was used as the cutoff point for chronic kidney disease (CKD).

Statistical analyses were performed using the Predictive Analytics Software statistics for Windows, v21.0 (SPSS Inc, Chicago, IL). Normal distribution of data was assessed by the Kolmogorov-Smirnov test and data were presented as mean (±SD) or as median (interquartile range) where appropriate. Categorical variables were presented as absolute frequency and percentage. When analyzing baseline eGFR, the Mann-Whitney *U* test was used to make comparisons.

Agreement between the CKD-EPI and MDRD equations was assessed using the general linear model. The Bland and Altman method was used to estimate the global bias throughout the mean difference between GFR values and plot the differences against their means. The Kappa index was used to analyze the level of agreement between the NKF stage classifications obtained using CKD-EPI and MDRD. Statistical significance was considered if the *p*-value <0.05.

## Results

Of the 4622 eligible patients with follow-up in 2014, 4503 were included for analysis. Excluded patients (six patients were diagnosed with end stage renal disease before HIV-infection, and the remaining 113 patients had no laboratory data in 2014) were less likely to be smokers, had a shorter duration of HIV infection (7.7 vs. 12.3 years), were less likely to be on antiretroviral treatment (91.5% vs. 97.3%), and had higher nadir CD4 counts and lower viral loads (data not shown).

### Demography

Demographic characteristics are depicted in Table 1. Median age was 45 years [interquartile range (IQR) 37–52], median body mass index was 22.9 kg/m^2^ (IQR 21.2–24.9), and median “baseline” creatinine was 0.93 mg/dL (IQR 0.82–1.05). Ethnicity was available for 2/3 of the patients; 98% of them were non-black. History of hypertension, diabetes, and dyslipidemia was noted in 14, 5, and 13% of patients, respectively, and 5% of the patients had prior cardiovascular diseases. Median duration of HIV infection was 11 years (IQR 5–19), 20% met the clinical definition for AIDS; 4365 patients (97%) were receiving combined ARV, and 89% had the status of virological suppression under treatment.

**Table 1:** Main demographic characteristics of 4503 patients with HIV infection included in the study

Variable	Estimates	n	Missing information
Median age, years (IQR)	45 (37–52)	4503	0
Female sex, n (%)	864 (19)	4503	0
Black race, n (%)	58 (2)	2879	1624
Body mass index, kg/m^2^ (IQR)	22.9 (21.2–24.9)	1394	3109
Current smoker, n (%)	2110 (51)	4154	349
Hypertension, n (%)	619 (14)	4503	0
Diabetes mellitus, n (%)	225 (5)	4503	0
Dyslipidemia, n (%)	596 (13)	4503	0
Cardiovascular disease, n (%)	202 (5)	4503	0
Hepatitis B coinfection, n (%)	149 (3)	4485	18
Hepatitis C coinfection, n (%)	1006 (22)	4495	8
Mode of HIV acquisition, n (%)^a^		4503	0
Homo/bisexual	2650 (62)		
Heterosexual	1176 (28)		
Injection drug use	641 (15)		
Other/Unknown route	290 (6)		
Duration of HIV infection, years (IQR)	11.3 (5.1–19)	4478	25
Prior AIDS (yes), n (%)	900 (20)	4503	0
Current ART regimen		4503	0
No treatment	138 (3)		
NNRTI-based	2381 (53)		
PI-based	144 (3)		
Boosted PI-based	1315 (29)		
Integrase inhibitors-based	493 (11)		
Others	32 (1)		
Nadir CD4 cell count (IQR)	249 (129–369)	4484	19
Current CD4 cell count (IQR)	628 (450–830)	4501	2
Current HIV infection status		4503	0
Suppression on Treatment	4031 (90)		
No suppression (viral load > 50 copies)	471 (10)		

*IQR*, interquartile range; *HIV*, human immunodeficiency virus; *AIDS*, acquired immunodeficiency syndrome; *NNRTI*, non-nucleoside reverse transcriptase inhibitor; *PI*, protease inhibitor

^a^Patients can present more than one HIV transmission route

### GFR evaluation

Overall baseline median eGFR was 95.2 mL/min/1.73 m^2^ and 90.4 mL/min/1.73 m^2^ by the CKD-EPI and MDRD creatinine equations, respectively (*p* < 0.001).

In the absence of a gold standard method for estimating GFR, we considered two models of comparison: using CKD-EPI as reference and then using MDRD as reference measure. When considering only patients with eGFR ≥60 mL/min/1.73 m^2^ and CKD-EPI estimation as reference, baseline mean eGFR was 95.12 mL/min/1.73 m^2^ and 92.99 mL/min/1.73 m^2^ by CKD-EPI and MDRD equations, respectively (*p* < 0.001). When using the MDRD estimation as reference, baseline mean eGFR was 95.21 mL/min/1.73 m^2^ and 93.11 mL/min/1.73 m^2^ by CKD-EPI and MDRD creatinine equations, respectively (*p* < 0.001). When considering only patients with eGFR <60 mL/min/1.73 m^2^ and CKD-EPI estimation as reference, baseline mean eGFR was 48.08 mL/min/1.73 m^2^ and 48.60 mL/min/1.73 m^2^ by CKD-EPI and MDRD equations, respectively (*p* = 0.999). When using MDRD estimation as reference, baseline mean eGFR was 48.79 mL/min/1.73 m^2^ and 48.83 mL/min/1.73 m^2^ by CKD-EPI and MDRD equations, respectively (*p* = 0.999).

Using the two consecutive measures of serum creatinine, the classification of patients for stages of CKD is presented in Table 2. When using the CKD-EPI formula as reference, 1091 (24.2%) patients presented mild reduced GFR, 118 (2.6%) patients had stage 3 CKD, and 5 (0.11%) patients presented stage 4 CKD. When using the MDRD formula as reference, 1396 (31%) patients presented mild reduced GFR, 116 (2.6%) patients had stage 3 CKD, and 2 (0.04%) patients presented stage 4 CKD. There were 19 patients under renal substitutive therapy at baseline (11 on dialysis and 8 kidney transplant recipients), classified as stage 5 CKD. However, the MDRD equation has not been validated for normal GFR, and is it not possible to discriminate between GFR > 90 mL/min/1.73 m^2^ and between 60 and 89 mL/min/1.73 m^2^; they should be reported as “≥60 mL/min/1.73 m^2^”. On the contrary, CKD-EPI can report these two stages separately.

**Table 2:**
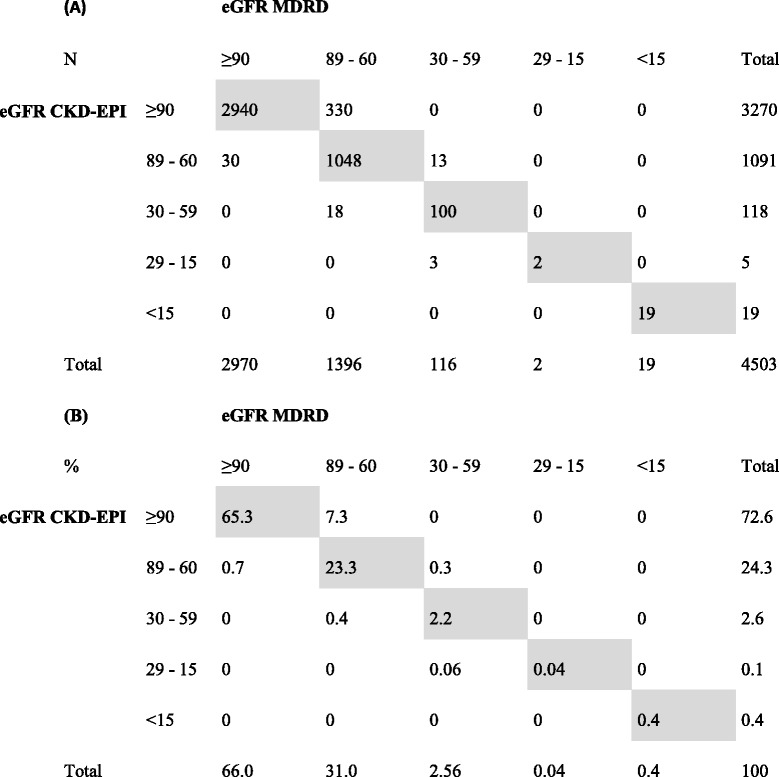
Grades of renal impairment according the National Kidney Foundation guidelines. Comparison of classifications provided using CKD-EPI and MDRD equations^a^. Absolute frequencies (A) and percentages (B) of the studied population

^a^Shaded cells indicate agreement between CKD-EPI and MDRD-derived eGFR estimations

### CKD-EPI versus MDRD equations

A substantial agreement was found between the MDRD and CKD-EPI creatinine formulae. When evaluating the absolute measures of eGRF, there was a similar distribution both in the baseline measure (*p* = 0.548) and in the second measure (*p* = 0.259).

**Fig. 1:**
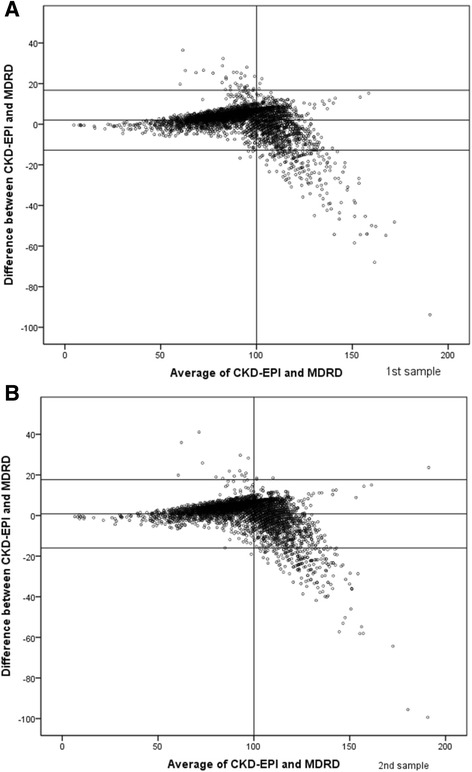
Bland Altman plot of agreement between MDRD and CKD-EPI equations for baseline measure (**a**) and second measure of creatinine (**b**)

Figure 1 shows a Bland-Altman plot of the agreement between the MDRD and CKD-EPI equations for the baseline measure and second measure of creatinine. Overall, the mean (SD) difference between the estimates from the two formulae was 2.003, with limits of agreement of 16.753 to -12.753 for the baseline measure, and 0.8232, with limits of agreement of 17.636 to -15.9896, for the second measure of creatinine.

Of the 4503 measurements, 4109 (91.2%) agreed, that is, they were classified as normal, mildly decreased, moderately decreased (stage 3), severely decreased (stage 4) or end-stage renal insufficiency (stage 5) with both formulae, with a kappa index of 0.803. However, 7.3% of patients were classified as having “mild renal impairment” with the MDRD equation but as having “normal renal function” with the CKD-EPI equation.

## Discussion

In this large sample of HIV-infected patients, there was a good correlation between the CKD-EPI and MDRD equations. The CKD-EPI equation gave somewhat higher eGFR estimates than the MDRD formula (95.2 mL/min/1.73 m^2^ and 90.4 mL/min/1.73 m^2^ by CKD-EPI and MDRD creatinine equations, respectively; *p* < 0.001), resulting in smaller but non-significant numbers of patients with eGFR <90 mL/min/1.73 m^2^ (27.4% vs. 34%). Although the clinical relevance of the difference found should be interpreted with caution, our data are consistent with observations in the general [19, 20] and HIV-infected populations [21], suggesting that MDRD may overestimate the severity of renal impairment. Studies in the general population comparing CKD-EPI and MDRD against isotopic GFR observed less bias, improved precision and greater accuracy with CKD-EPI [22], although others have found lesser mean bias with MDRD [23].

In this analysis, at least a quarter of patients were diagnosed with mild reduced GFR disease (eGFR 60–89 mL/min/1.73 m^2^). This is important in detecting subclinical renal disease and identifying those patients with a higher cardiovascular risk or at risk of developing declining renal function. In practical terms, detecting minor changes in eGFR allows the early adoption of lifestyle modification and medication therapies, as appropriate, to control hypertension, dyslipidemia and hyperglycemia. In addition, nephrotoxic medications should be avoided, and medication doses (particularly when prescribing ARV) must be adjusted for renal function. For example, Stribild® (combination of emtricitabine, tenofovir disoproxil fumarate, cobicistat and elvitegravir for a complete 1-pill, once-a-day HIV-1 treatment) prescription information contraindicates the use of this drug in patients with eGFR below 70 mL/min/1.73 m^2^ [24]. The MDRD equation misdiagnosed patients with apparent ‘normal’ renal function (over 60 mL/min/1.73 m^2^).

Chronic kidney disease stages III-V (eGFR <60 mL/min/1.73 m2) were found in 3% of HIV-infected patients with both equations, a prevalence similar to the EuroSIDA study and the French [25, 26] cohorts. However, this prevalence was lower than that found in a recent American analysis, in which 7.5% of HIV-infected patients had CKD [27], and even lower than a Nigerian analysis [28] that showed 52.6% of patients with GFR <60 mL/min. Possible explanations are the higher proportions of blacks and hypertensive patients on American and Nigerian cohorts, the duration of untreated HIV infection, the length of exposure to nephrotoxic antiretrovirals, as well as lifestyle-related variables and socioeconomic factors reported to impact health care coverage [29].

This study has some limitations: first, its retrospective nature; second, the absence of a direct measure of glomerular filtration rate for comparison; third, the unavailability of urine protein/creatinine ratios; and, finally the absence of an outcome measure for those patients with a reduced eGFR, which fell outside the scope of this study.

In summary, the evaluation of renal function is essential for the clinical management of patients with HIV-infection. In line with observations in the general population, this study suggests that the MDRD equation may underestimate renal function in subjects with GFRs over 60 mL/min/1.73 m^2^. The main contribution of this report was that the CKD-EPI equation identified 24% of patients with mild reduced eGFR, not identified using the MDRD equation. This finding has strong implications for clinical practice, as it provides a framework for identifying patients at risk of poorer outcomes (overt chronic kidney disease, cardiovascular disease and mortality) and allows better clinical management, especially in terms of the dose adjustment of antiretroviral drugs.

## Conclusion

In conclusion, the CKD-EPI equation must be considered as the first-choice method to evaluate renal function in patients with HIV-infection.

## Abbreviations

ARV: Antiretroviral therapy
CG: Cockroft-gault
CKD: Chronic kidney disease
CKD-EPI: CKD epidemiology collaboration
eGFR: Estimated glomerular filtration rate
GFR: Glomerular filtration rate
HIV: Human immunodeficiency virus
IQR: Interquartile range
K/DOQI: US national kidney foundation’s kidney disease outcome quality initiative
MDRD: Modification of diet in renal disease
NKF: National kidney foundation
SD: Standard deviation
